# Effects of grazing intensity on diversity and composition of rhizosphere and non‐rhizosphere microbial communities in a desert grassland

**DOI:** 10.1002/ece3.10300

**Published:** 2023-07-10

**Authors:** Yang Yang, Hao Zhang, Wei Liu, Jiamei Sun, Mengli Zhao, Guodong Han, Qingmin Pan

**Affiliations:** ^1^ State Key Laboratory of Vegetation and Environmental Change Institute of Botany, the Chinese Academy of Sciences Beijing China; ^2^ University of Chinese Academy of Sciences Beijing China; ^3^ Inner Mongolia Agriculture University College of Grassland, Resources and Environment Hohhot China

**Keywords:** microbial composition, microbial diversity, non‐rhizosphere soil, rhizosphere soil‐grazing intensity

## Abstract

Overgrazing‐induced grassland degradation has become a serious ecological problem worldwide. The diversity and composition of soil microbial communities are sensitive to grazing disturbances. However, our understanding is limited with respect to the effects of grazing intensity on bacterial and fungal communities, especially in plant rhizosphere. Using a long‐term grazing experiment, we evaluated the diversity and composition of microbial communities in both rhizosphere and non‐rhizosphere soils under three grazing intensities (light, moderate, and heavy grazing) in a desert grassland and examined the relative roles of grazing‐induced changes in some abiotic and biotic factors in affecting the diversity and composition of microbial communities. Our results showed that soil bacteria differed greatly in diversity and composition between rhizosphere and non‐rhizosphere zones, and so did soil fungi. Moderate and heavy grazing significantly reduced the rhizosphere bacterial diversity. Grazing intensity substantially altered the bacterial composition and the fungal composition in both zones but with different mechanisms. While root nitrogen and soil nitrogen played an important role in shaping the rhizosphere bacterial composition, soil‐available phosphorus greatly affected the non‐rhizosphere bacterial composition and the fungal composition in both soils. This study provides direct experimental evidence that the diversity and composition of microbial communities were severely altered by heavy grazing on a desert grassland. Thus, to restore the grazing‐induced, degraded grasslands, we should pay more attention to the conservation of soil microbes in addition to vegetation recovery.

## Graphical abstract

Our study found that grazing in fragile ecosystems had a stronger effect on rhizosphere soil microorganisms than in non‐rhizosphere soil. The restoration and protection of rhizosphere microbial community should be emphasized when considering the restoration of degraded grassland.

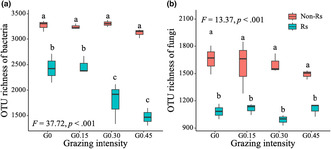

## 1 INTRODUCTION

Grasslands cover about 40% of the earth's land surface and are mainly used for livestock grazing (Suttie et al., 2005). While light and/or moderate grazing can improve grassland diversity and productivity, heavy grazing generally causes severe grassland degradation (Kemp et al., 2013; McNaughton, 1979). By far, overgrazing‐induced grassland degradation has become a major ecological problem worldwide (Dlamini et al., 2016; Eldridge et al., 2017; van de Koppel et al., 1997). This is especially the case in the desert steppe ecosystems on the Mongolian Plateau, where the vegetation cover is ca. 25% and the annual biomass production is ca. 130 g m^−2^ (Kemp et al., 2013; Wei et al., 2013).

Overgrazing not only reduces vegetation cover, decreases plant production, and alters vegetation composition (Wei et al., 2013) but also has profound impacts on the diversity and composition of soil microbial communities (Hu et al., 2017; Lamb et al., 2011; Wang, Ding, et al., 2021; Zhang et al., 2020). Some studies in desert steppe ecosystems showed that grazing could significantly reduce the diversity of microbial communities in bulk soil and in rhizosphere soil (Wang, Ding, et al., 2021; Zhang et al., 2019). In contrast, another study in the same ecosystem showed that grazing had little effect on bacterial diversity and fungal diversity in rhizosphere soil, nor in bulk soil (Chen et al., 2020). These inconsistent results suggest that the impacts of grazing on microbial communities are likely to be mediated by grazing intensity and grazing history. Moreover, a previous study indicated that grazing could alter the composition of microbial communities in rhizosphere soil rather than in non‐rhizosphere soil (Zhang et al., 2019), highlighting the contrasting responses of microbial communities between rhizosphere and non‐rhizosphere soils. In fact, soil microbial communities can differ greatly in diversity and composition between the two zones because host plants usually exert a select effect on microbial taxa in their rhizosphere soil (Hartmann et al., 2009; Nan et al., 2020). Considering that grazing has different impacts on different plant species and that different plant species prefer different microbial taxa, the impacts of grazing on rhizosphere microbial communities are expected to be stronger than on non‐rhizosphere microbial communities. However, how grazing intensity affects the soil bacterial and fungal communities in rhizosphere and non‐rhizosphere zones, especially over a decade, remains poorly understood.

Grazing can influence the soil microbe through several mechanisms. First, herbivory on plants can have dual effects on soil microbial communities. On the one hand, removal of aboveground biomass decreases the input of litter into the soil, which reduces the supply of carbon resource to the soil microbe, and thus causes a reduction in soil microbial richness (Dwivedi et al., 2017). On the other hand, removal of aboveground parts can also stimulate the allocation of organic nutrients to belowground organs (Mueller et al., 2017), resulting in enrichment of root exudates (Hamilton & Frank, 2001), which in turn affects the structure of microbial community (Hu et al., 2018). Second, manure from livestock can affect soil pH values (Wang, Ding, et al., 2021; Yang, Niu, et al., 2018; Yang, Zhu, et al., 2018) and improve the available soil nitrogen (Han et al., 2008). As a result, the relative abundance of copiotrophic taxa was enhanced, while that of oligotrophic taxa was reduced (Nie et al., 2018). Finally, livestock trampling decreases soil moisture due to increased soil compactness (Kobayashi et al., 1997), which in turn affects soil microbial properties (Chen et al., 2019). These studies provide important insights into how grazing‐induced changes in biotic and abiotic factors can influence microbial communities; however, the relative roles of biotic and abiotic factors in affecting microbial responses to grazing intensity in rhizosphere and non‐rhizosphere soils are poorly evaluated.

Given that desert steppe is more sensitive to grazing disturbance than other steppes (e.g., typical steppe or meadow steppe) due to its relatively lower precipitation, lower vegetation cover, and lower biomass productivity (Wei et al., 2013), we addressed these research gaps by a 15‐year grazing intensity experiment in a desert steppe. We focused on three specific questions: (i) How do microbial communities differ in diversity and composition between rhizosphere and non‐rhizosphere? (ii) How do the bacterial and fungal communities respond to grazing intensities? (iii) What are the relative roles of abiotic and biotic factors in affecting the responses of bacterial and fungal communities to grazing intensity? Overall, this study contributes to our understanding of the impacts of grazing intensity on the diversity and composition of microbial communities in rhizosphere and non‐rhizosphere soils, as well as the mechanisms driving these changes.

## 2 MATERIALS AND METHODS

### 2.1 Study site

This study was carried out in a desert grassland on the Inner Mongolia Plateau (41°46′43.6″ N, 111°53′41.7″ E; elevation 1456 m). This area has a temperate continental grassland climate, with an annual mean temperature and precipitation of 3.6°C and 214 mm, respectively (Wang et al., 2014). About 80% of precipitation occurs during the growing season (May to September). The vegetation at the experiment site was dominated by *Stipa breviflora* and *Cleistogenes songorica*. The soil is a Kastanozem soil (FAO soil classification) with a sandy loam texture. The Siziwang Banner, Inner Mongolia Autonomous Region of China, is traditionally used as pastures for free grazing and 90% of them have been degraded due to overgrazing (Kemp et al., 2013).

### 2.2 Experimental design

In 2004, a long‐term grazing intensity experiment was established. We had four grazing intensities: control (zero sheep‐unit ha^−1^ month^−1^, G0), light (0.15 sheep‐unit ha^−1^ month^−1^, G0.15), moderate (0.30 sheep‐unit ha^−1^ month^−1^, G0.30), and heavy grazing (0.45 sheep‐unit ha^−1^ month^−1^, G0.45; Zhang et al., 2021). G0.15, G0.30, and G0.45 were approximately corresponding to 18%, 40%, and 60% of annual biomass consumed by animals, respectively. Each treatment had three replicates. Under a complete randomized block design, 12 plots, each with an area of 4.4 ha, were separated by fences. Sheep of similar size were selected in advance and the grazing period lasted from June 1 to November 30 every year. The plots were grazed from June 1 to November 30 every year and from 6 AM to 6 PM every day. At night, the sheep were placed in an enclosure.

### 2.3 Field sampling and laboratory analysis

We randomly selected 30 bunches of *C. songorica* in each plot in late August 2018, corresponding to the peak biomass time. Each bunch of plants together with soil attached to their roots were dug out to a depth of 15 cm (Cao et al., 2019; Yang, Niu, et al., 2018; Yang, Zhu, et al., 2018), with approximately 90% of the roots harvested. Non‐rhizosphere soil was defined as those shaken from the roots while rhizosphere soil was defined as those that adhere to the roots tightly and cannot be shaken off (Chaudhary et al., 2015). After a bunch of plants with soil was dug out, we shook the plants to collect the non‐rhizosphere soil that departed from the plant roots until no soil could be shaken off. Then we brushed the roots to collect the rhizosphere soil until no soil could be brushed off (Chaudhary et al., 2015; Chen et al., 2020; Fu et al., 2022). The plants of *C. songorica* were separated into shoots and roots and oven‐dried at 65°C to a constant weight. Soil samples from the same plot were mixed to form a composite sample; therefore, we obtained 12 rhizosphere and 12 non‐rhizosphere composite soil samples (four treatments × thee replications).

Each composite soil sample was equally separated into three portions: one air‐dried, one kept fresh at 4°C, and one stored at −80°C. The fresh sample was used to determine soil moisture and inorganic nitrogen concentration. Soil moisture was measured using the gravimetric method. The weighted fresh soil (approximately 20 g) was oven‐dried at 105°C to a constant weight. Soil moisture was determined by the difference in weight between fresh soil and oven‐dried soil. Concentrations of ammonium nitrogen and nitrate nitrogen were measured using a continuous flow analyzer (AutoAnalyzer III; Bran+Luebbe), and the inorganic nitrogen was their sum. The air‐dried samples were used to determine the concentrations of total carbon, total nitrogen, total phosphorus, available phosphorus, and pH values. Concentrations of total carbon and total nitrogen in soil/root samples were determined by an element analyzer (Elementar Vario Micro Cube). The air‐dried soil samples were digested with a mixture of H_2_SO_4_‐H_2_O_2_. Then the concentration of soil total phosphorus was determined by the molybdenum blue‐ascorbic acid method on a spectrophotometer. The available phosphorus was determined by the same methods after extraction with 0.5 M NaHCO_3_. Soil pH was measured using a pH meter. The frozen samples were used to extract DNA for evaluating the richness and composition of microbial communities.

### 2.4 DNA extraction, PCR amplification, and sequencing

For each sample, DNA was extracted from 0.25 g of thawed soil using a DNeasy® PowerSoil® DNA isolation kit (Qiagen). The quality of isolated DNA was determined by electrophoresis on 1% agarose gel. The purity was examined using NanoDrop. In total, 12 rhizosphere and 12 non‐rhizosphere DNA samples were prepared.

The 16S rRNA gene was amplified to estimate the relative abundance of soil bacteria while internal transcribed spacer (ITS) region for the fungi. The paired primers, 341F and 806R, were used to target the hypervariable V3 + V4 region of the 16S rRNA gene; while the paired primers, ITS5‐1737F and ITS2‐2043R, were used for the ITS1 regions of fungi. The primers in each sample were tagged with unique barcodes. All PCRs were carried out in 30‐μL reactions with 10 ng of template DNA, 0.2 μM of forward and reverse primers, and 15 μL of Phusion® High‐Fidelity PCR Master Mix (New England Biolabs). For thermal cycles, the initial denaturation was at 98°C for 1 min, followed by 30 cycles of denaturation at 98°C for 10 s. Then annealing was at 55°C for 30 s and elongation at 72°C for 30 s. Finally, an extension was at 72°C for 5 min.

The PCR products of each sample and 1× loading buffer (with SYBR green) were mixed with equal volumes and then electrophoresed on a 2% agarose gel to examine the successful amplification of target DNA fragments. The PCR products for each sample were quantified and mixed with 80 ng of DNA in equal proportions. The resulting mixed PCR products were electrophoresed with 2% agarose gel, and the DNA fragments ranging from 400 to 450 bp were excised and then purified with a GeneJET™ Gel Extraction Kit (Thermo Scientific).

Following the manufacturer's recommendation, the Ion Plus Fragment Library Kit 48 rxns (Thermo Scientific) was used for sequencing libraries preparation. The Qubit@ 2.0 Fluorometer (Thermo Scientific) and the Agilent Bioanalyzer 2100 system (Agilent) were used to evaluate the quality of the library. The libraries were sequenced on the Ion S5™XL platform, generating 400‐bp/600‐bp single‐end reads.

### 2.5 Bioinformatics analyses

Based on the unique barcode of each sample, single‐end reads were identified and truncated by shearing off the barcode and primer sequences. The Cutadapt quality‐controlled process was used to filter the raw reads under specific filtering conditions (Martin, 2011), we obtained the high‐quality clean reads. The reads were compared with the reference database (Silva and Unite database) using the UCHIME algorithm to detect chimera sequences (Edgar et al., 2011; Kõljalg et al., 2013; Quast et al., 2013) and remove them. Then, final clean reads were generated. We performed sequence analysis using Uparse software (Uparse v7.0.1001; Edgar, 2013). Sequences with a similarity >97% were assigned to the same operational taxonomic unit (OTU). The representative sequence for each OTU was screened for further annotation. The taxonomic information of bacterial representative sequences was annotated using the SILVA132 SSU rRNA database on Mothur algorithm (Quast et al., 2013). For fungal representative sequences, we annotated taxonomic information using the UNITE (v7.2) database‐based BLAST algorithm (Kõljalg et al., 2013), which was calculated by QIIME software (Version 1.9.1).

In total, we obtained 947,987 and 958,264 bacterial high‐quality sequences from rhizosphere and non‐rhizosphere samples, respectively. These reads were clustered into 23,160 and 37,278 OTUs. For fungi, we obtained 961,649 and 928,290 high‐quality sequences from rhizosphere and non‐rhizosphere samples, respectively, which were clustered into 11,695 and 17,424 OTUs (Table A1).

### 2.6 Statistical analysis

All statistical analyses were conducted in R 4.0.2 (R Core Team, 2020). We used the OTU richness, Shannon diversity, ACE, Chao1, and phylogenetic diversity of bacterial and fungal communities to evaluate the response of microbial diversity. Before analyses, Shapiro–Wilk test was used for normal distribution evaluation, and Bartlett's test for variance homogeneity of the data. Differences in bacterial and fungal diversity between rhizosphere and non‐rhizosphere zones through the Wilcoxon rank‐sum test, performed using the wilcox.test function in the package ‘stats’ (Field et al., 2012). Difference in composition (e.g. the relative abundance of main bacterial and fungal phylum or class) between rhizosphere and non‐rhizosphere zones was determined by the Wilcoxon rank‐sum test. Furthermore, to identify significant differences in bacterial and fungal taxa between rhizosphere and non‐rhizosphere zones, we used the linear discriminant analysis (LDA) effect size (LEfSe) method (Segata et al., 2011). The LEfSe analysis applied the Kruskal–Wallis sun‐rank test to detect taxa with significantly different abundances between rhizosphere and non‐rhizosphere zones and signed LDA log‐score (LDA ≥ 4 in this study) to estimate the effect size of each different abundance taxon (Segata et al., 2011).

One‐way analysis of variance (ANOVA) was used to assess the effects of grazing intensity on microbial diversity, composition, biotic variables (shoot biomass, root biomass, root total carbon, and root total nitrogen of *C. songorica*), and abiotic variables (concentrations of total carbon, total nitrogen, and total phosphorus, concentrations of available nitrogen and available phosphorus, soil pH, and soil moisture). After ANOVA, the Bonferroni test was used to perform a multiple comparison test that examined the differences in microbial diversity, composition, biotic and abiotic variables between treatments. Differences in microbial community composition between the two zones were evaluated using permutational multivariate analysis of variance (PERMANOVA) with the function “adonis” in the package “vegan” with 999 permutations (Oksanen et al., 2020), and visualized by Principal Coordinate Analysis (PCoA). The dissimilarity in microbial community composition was determined by the Bray–Curtis distance between treatments, performed with the “vegdist” function in package “vegan” (Oksanen et al., 2020).

A Mantel test was used to evaluate how changes in microbial community diversity or composition are related to biotic and abiotic variables by the “mantel test” function in the package “vegan”, and visualized by the package “linkET” (Huang, 2021). The multicollinearity of the variables was assessed based on variance inflation factors (VIF), and those with a VIF value <10 were regarded as variables with low collinearity. The relative importance of variables in affecting microbial richness and composition was evaluated with the package of “rdacca.hp” (Lai et al., 2021).

## 3 RESULTS

### 3.1 Microbial richness and composition in rhizosphere versus non‐rhizosphere

**FIGURE 1:**
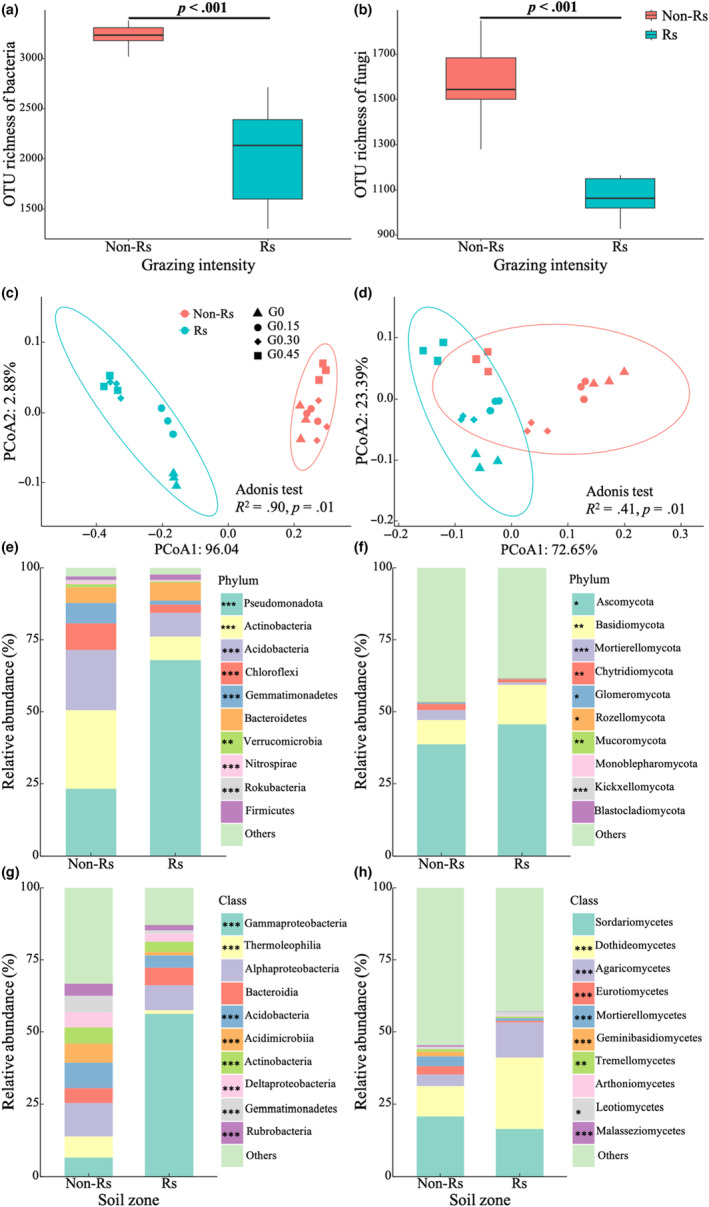
Comparisons in microbial community richness and composition between rhizosphere (Rs) and non‐rhizosphere (Non‐Rs) soils. *Note*: Bacterial (a) and fungal (b) richness; Difference in bacterial (c) and fungal (d) community composition; Relative abundance of main bacterial (e) and fungal (f) taxa at phylum level; Relative abundance of main bacterial (g) and fungal (h) taxa at class level. The taxa with a relative abundance <1% were assigned into “Others.” *, **, and *** indicated significant difference between rhizosphere and non‐rhizosphere soils by *t*‐test at *p* = .05, .01, and .001, respectively.

Microbial communities differed markedly between rhizosphere and non‐rhizosphere soils in OTU richness (Figure 1). The bacterial richness was significantly lower in rhizosphere than in non‐rhizosphere (Figure 1a), and so was the fungal richness (Figure 1b).

The bacterial community composition differed greatly between the two zones (Figure 1c). This was more evident in the relative abundances of three dominant bacterial phyla, Pseudomonadota, Acidobacteria, and Actinobacteria. The relative abundance of Pseudomonadota was 74.4% in rhizosphere but decreased to 23.6% in non‐rhizosphere (Figure 1e; Table A2). In contrast, the relative abundances of Acidobacteria and Actinobacteria were 6.4% and 5.9% in rhizosphere but increased to 21.2% and 26.7% in non‐rhizosphere, respectively (Figure 1e; Table A2). Similarly, at the class level, the relative abundance of Gammaproteobacteria was 6.6% in non‐rhizosphere, while it increased to 59.5% in rhizosphere (Figure 1g; Table A3). By contrast, the relative abundances of Thermoleophilia, Acidobacteria, Acidimicrobiia, Actinobacteria, Deltaproteobacteria, Gemmatimonadetes, and Rubrobacteria were significantly higher in non‐rhizosphere soil than in rhizosphere soil (Figure 1g; Table A3).

The fungal community composition also differed between the two zones (Figure 1d). For example, the relative abundance of Ascomycota was 48.8% in rhizosphere soil but decreased to 38.6% in non‐rhizosphere soil (Figure 1f; Table A2). At the class level, the relative abundances of Dothideomycetes and Agaricomycetes were 24.6% and 12.2% in rhizosphere soil, while decreased to 10.41% and 4.01% in non‐rhizosphere soil, respectively (Figure 1h; Table A3).

### 3.2 Effects of grazing intensity on microbial richness and composition

**FIGURE 2:**
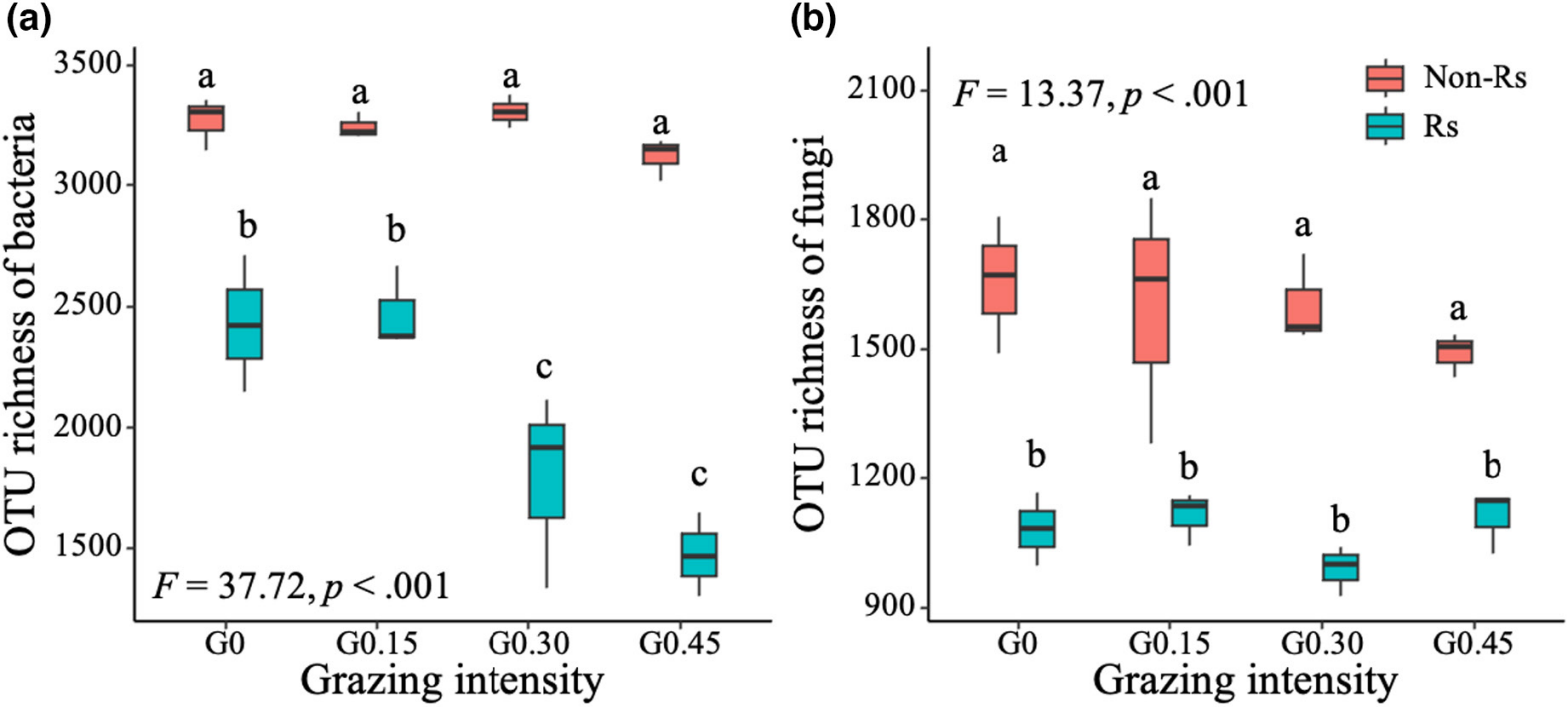
Effects of grazing intensity on microbial community richness in rhizosphere (Rs) and non‐rhizosphere (Non‐Rs) soils. *Note*: G0, G0.15, G0.30, and G0.45 refer to control, light, moderate, and heavy grazing, respectively. Different lowercase letters indicate significant difference between treatments at *p* = .05.

Grazing intensity had significant impacts on rhizosphere bacterial richness but not on that in non‐rhizosphere soil (Figure 2a). However, grazing intensity had little impact on fungal richness in both zones (Figure 2b).

**FIGURE 3:**
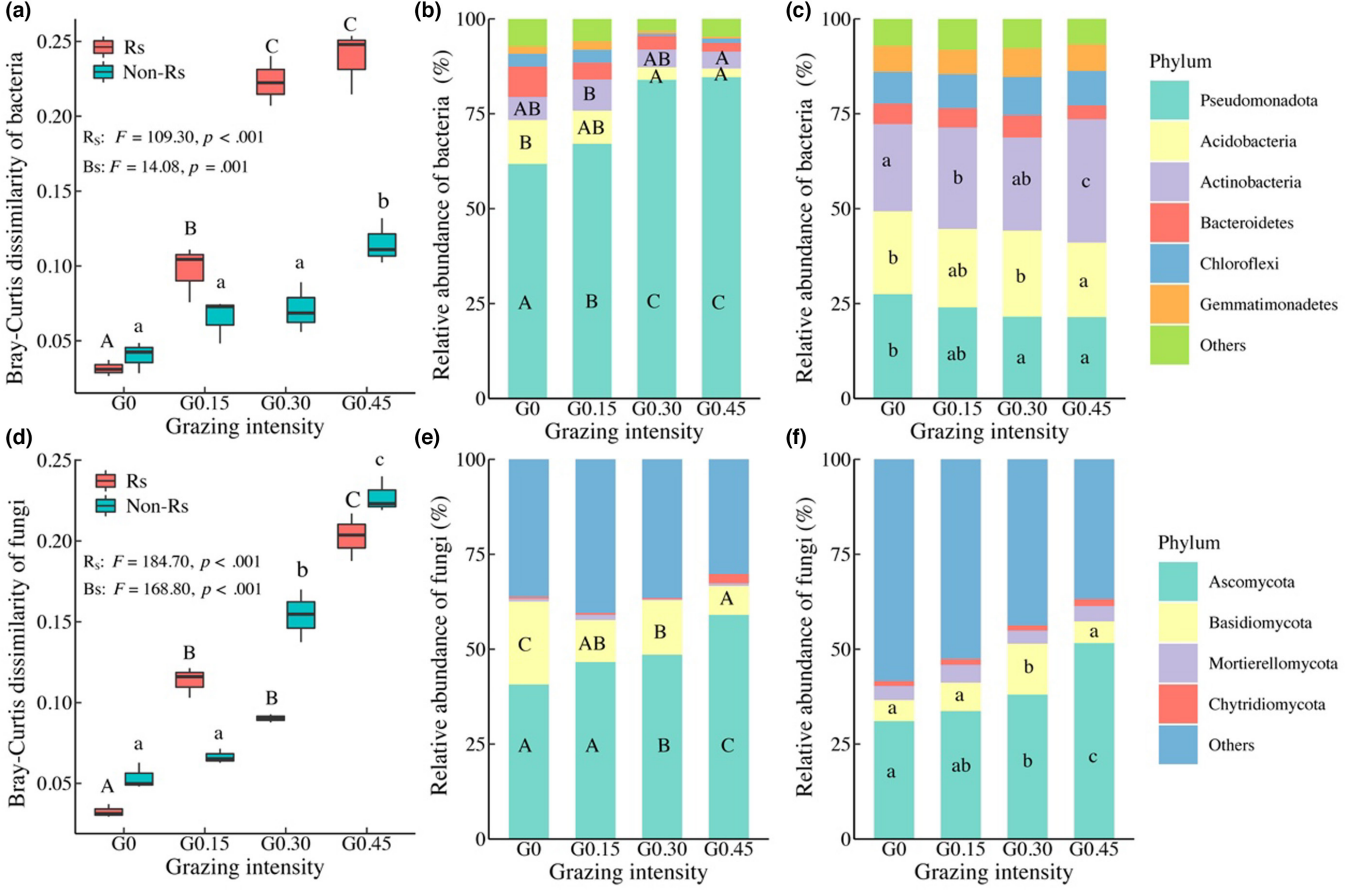
Effects of grazing intensity on the dissimilarity of microbial community composition and on the relative abundance of microbial taxa in rhizosphere (Rs) and non‐rhizosphere (Non‐Rs) soils. *Note*: The dissimilarity of bacterial communities (a) and the dissimilarity of fungal communities (d); The relative abundance of bacterial phyla in rhizosphere (b) and in non‐rhizosphere (c); Relative abundance of fungal phyla in rhizosphere (e) and in non‐rhizosphere (f). Different capital and lowercase letters indicate significant differences between treatments in non‐rhizosphere and rhizosphere soils, respectively. G0, G0.15, G0.30, and G0.45 are the same as in Figure 2.

For the microbial composition, the dissimilarity in the bacterial community exhibited an increasing trend with the increase in grazing intensity in both zones (Figure 3a). In rhizosphere soil, the relative abundance of Pseudomonadota increased significantly under moderate and heavy grazing while that of Acidobacteria decreased (Figure 3b; Table A4). In non‐rhizosphere soil, the relative abundances of Pseudomonadota and Acidobacteria decreased with heavy grazing, while that of Actinobacteria increased (Figure 3c; Table A4). The dissimilarity in the fungal community also displayed an increasing trend with the increase in grazing intensity in both zones (Figure 3d). Specifically, the dominant taxa, Ascomycota, showed a marked increase in relative abundance under moderate and heavy grazing (Figure 3e,f; Table A4).

### 3.3 Biotic and abiotic drivers for microbial richness

**FIGURE 4:**
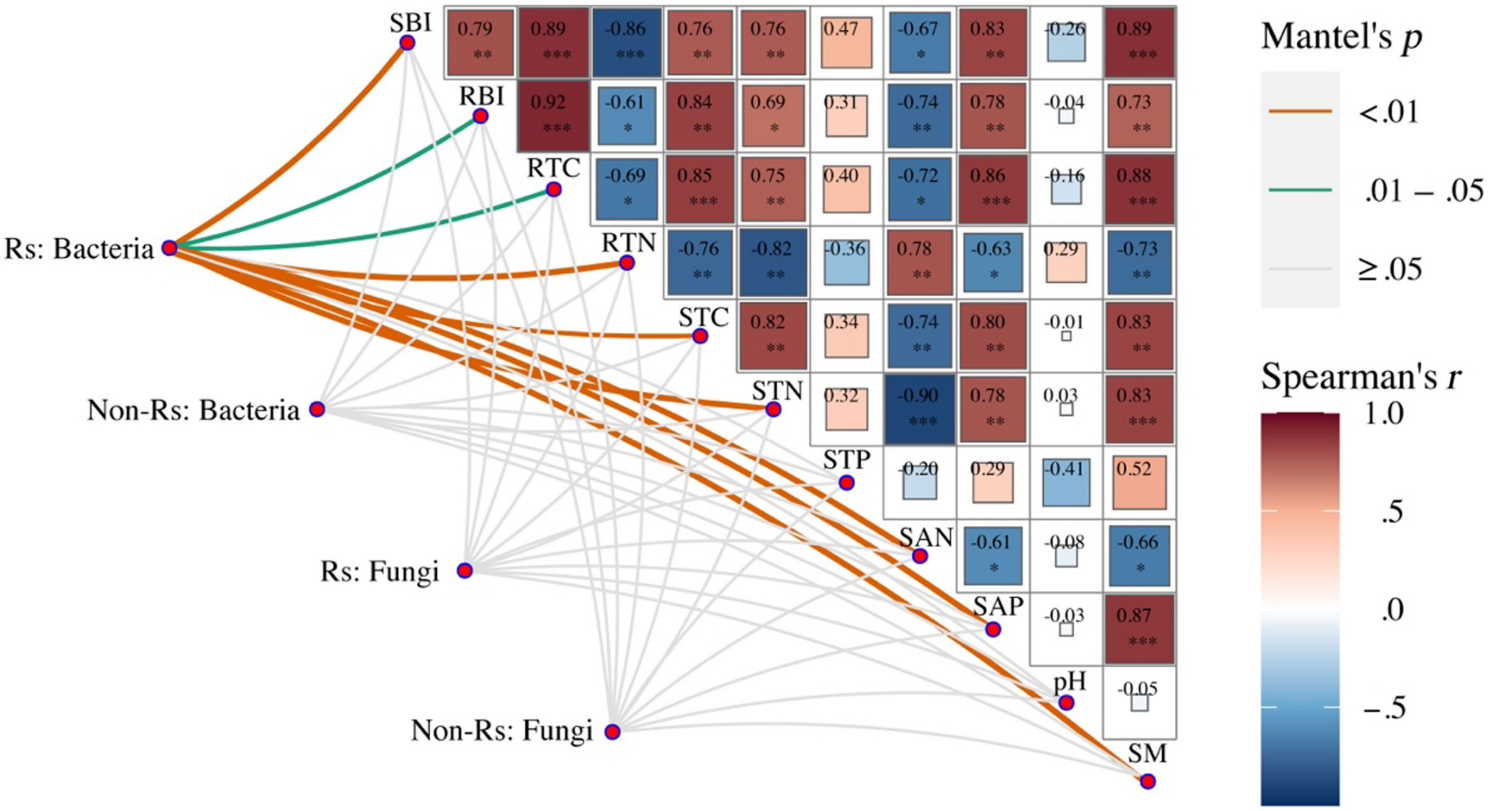
Impacts of abiotic and biotic factors on bacterial and fungal richness in rhizosphere (Rs) and non‐rhizosphere (Non‐Rs) soils. RBI, root biomass; RTC, root total carbon; RTN, root total nitrogen; SAN, soil‐available nitrogen; SAP, soil‐available phosphorus; SBI, shoot biomass; SM, soil moisture; STC, soil total carbon; STN, soil total nitrogen; STP, soil total phosphorus. Overall *R* ^2^ indicates the total explained fraction by the six variables.

In general, all the biotic and abiotic variables examined, except soil total phosphorus and soil pH, significantly affected soil bacterial richness in the rhizosphere, whereas that in the non‐rhizosphere was not affected by these variables (Figure 4). The fungal richness in both zones was not affected by examined biotic and abiotic variables (Figure 4).

### 3.4 Biotic and abiotic drivers for microbial community composition

**FIGURE 5:**
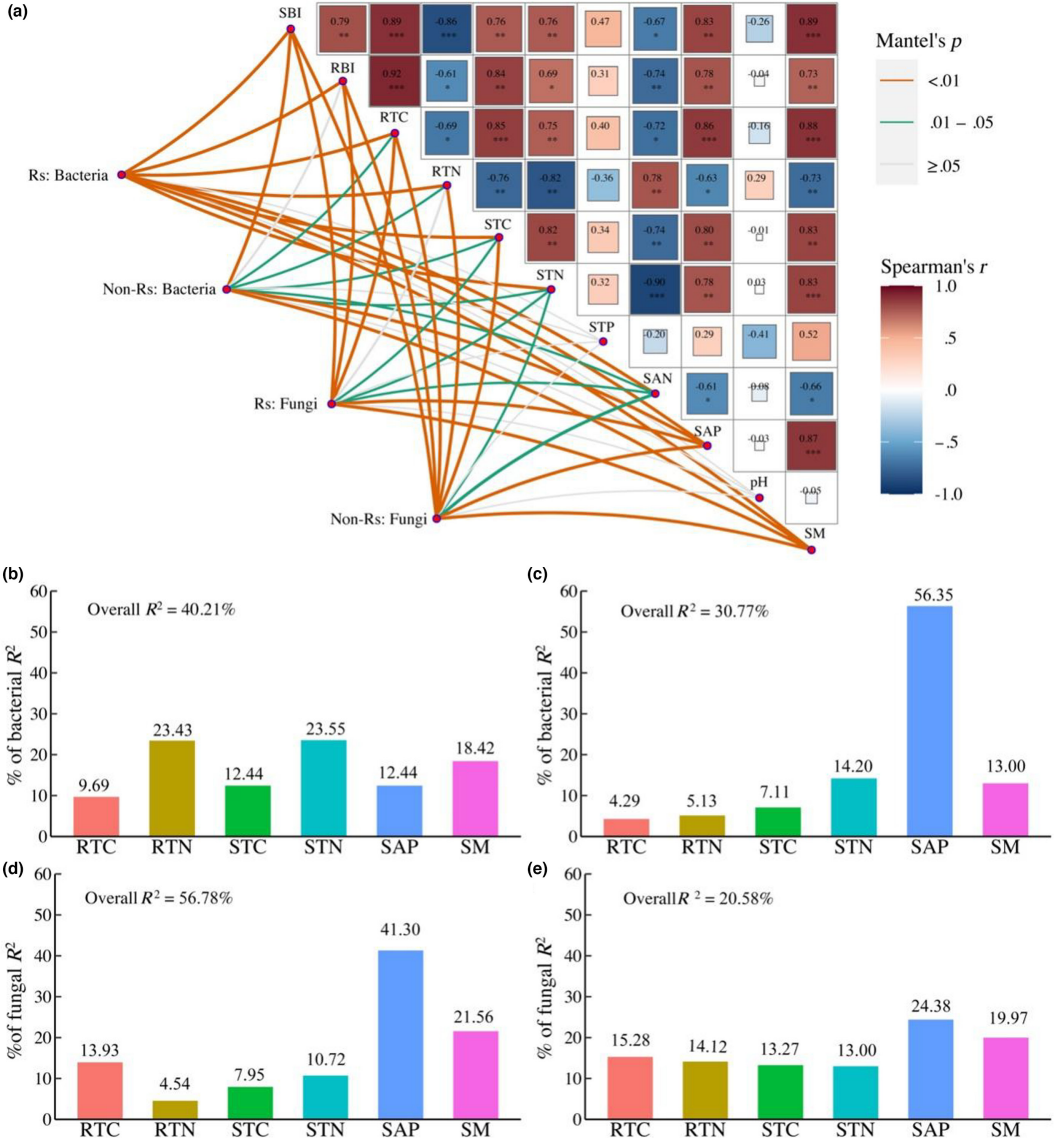
Impacts of abiotic and biotic factors on microbial compositions in rhizosphere (Rs) and non‐rhizosphere (Non‐Rs) soils. *Note*: Mantel results on the impacts of the variables examined on bacterial and fungal compositions (a). Relative roles of six variables with low collinearity on rhizosphere (b) and non‐rhizosphere bacterial compositions (c). Relative roles of six variables with low collinearity on rhizosphere (d) and non‐rhizosphere fungal compositions (e). SBI, RBI, RTC, RTN, STC, STN, STP, SAN, SAP, and SM are the same as in Figure 4. Overall *R* ^2^ indicates the total explained fraction by the six variables.

In general, all the biotic and abiotic variables examined, except soil total phosphorus and soil pH, affected the composition of the bacterial or fungal communities in both zones (Figure 5a). Moreover, among six variables with low collinearity, the rhizosphere bacterial composition was mainly affected by root total nitrogen and soil total nitrogen, whereas that in non‐rhizosphere was mainly affected by soil‐available phosphorus (Figure 5b,c). In addition, soil‐available phosphorus was consistently the primary variable affecting the fungal composition in both rhizosphere and non‐rhizosphere (Figure 5d,e).

## 4 DISCUSSION

### 4.1 Microbial diversity and composition in rhizosphere and non‐rhizosphere soils as influenced by grazing intensity

**FIGURE A1:**
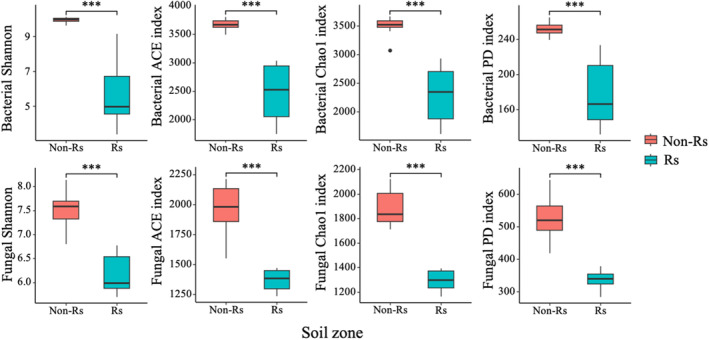
The comparison of soil bacterial and fungal diversity indices in rhizosphere and non‐rhizosphere soils. Non‐Rs, non‐rhizosphere soil; PD, phylogenetic diversity; Rs: rhizosphere soil. ***, *p* < .001.

Our results demonstrated that the microbial communities differed markedly between rhizosphere and non‐rhizosphere in diversity and composition in this desert steppe ecosystem. The microbial diversity indices in rhizosphere, including richness, Shannon diversity, ACE, Chao1, and phylogenetic diversity, were lower than those in non‐rhizosphere (Figures 1 and A1), which is in line with previous findings that microbial diversity decreased with decreasing distance to plant roots (Donn et al., 2015; Fan et al., 2017), likely because the rhizosphere microbiomes are derived from the non‐rhizosphere microbiomes under the selection effect of host plants (Hartmann et al., 2009; Nan et al., 2020).

The microbial composition also differed greatly between the two zones (Figure 1c,d). This was especially evident for the relative abundances of dominant microbial taxa. The relative abundance of Pseudomonadota, a dominant bacterial phylum, was 23.6% in non‐rhizosphere but increased to 74.4% in rhizosphere (Figure 1e; Table A2). Further LEfSe analysis indicated that the increased abundance of Pseudomonadota can be attributed to the enrichments in Gammaproteobacteria, Pseudomonadales, Pseudomonadaceae, *Pseudomonas*, *Pseudomonas frederiksbergensis* at class, order, family, genus and species levels in rhizosphere zone under light or heavy grazing intensity (Figure A4). Such changes are likely due to their similar strategy in nutrient use. Pseudomonadota is a representative copiotrophic bacteria phylum (Nie et al., 2018; Zhang et al., 2018, 2020). The increased availability of nutrients in rhizosphere due to acid activation by root exudates fosters its growth (Cesco et al., 2010), because more nutrients facilitate copiotrophic bacteria but restrict the oligotrophic bacteria according to the oligotroph–copiotroph hypothesis (Fierer et al., 2007; Leff et al., 2015). Similarly, Ascomycota is a dominant fungal phylum, and its relative abundance increased from 38.6% in non‐rhizosphere to 48.8% in rhizosphere (Figure 1f; Table A2). A similar result had been observed in an alpine grassland (Jiang et al., 2021). Our further analyses indicated that the higher abundance of Ascomycota in rhizosphere zone mainly stemmed from the higher abundances of Dothideomycetes and Agaricomycetes at the class level (Figure 1h). The relative abundances of Dothideomycetes and Agaricomycetes increased from 10.41% and 4.01% in the non‐rhizosphere soil to 24.55% and 12.16% in the rhizosphere soil, respectively (Table A3). Collectively, these results suggest that the microenvironment of the plant rhizosphere may facilitate the flourishing of certain bacterial and fungal taxa.

**FIGURE A2:**
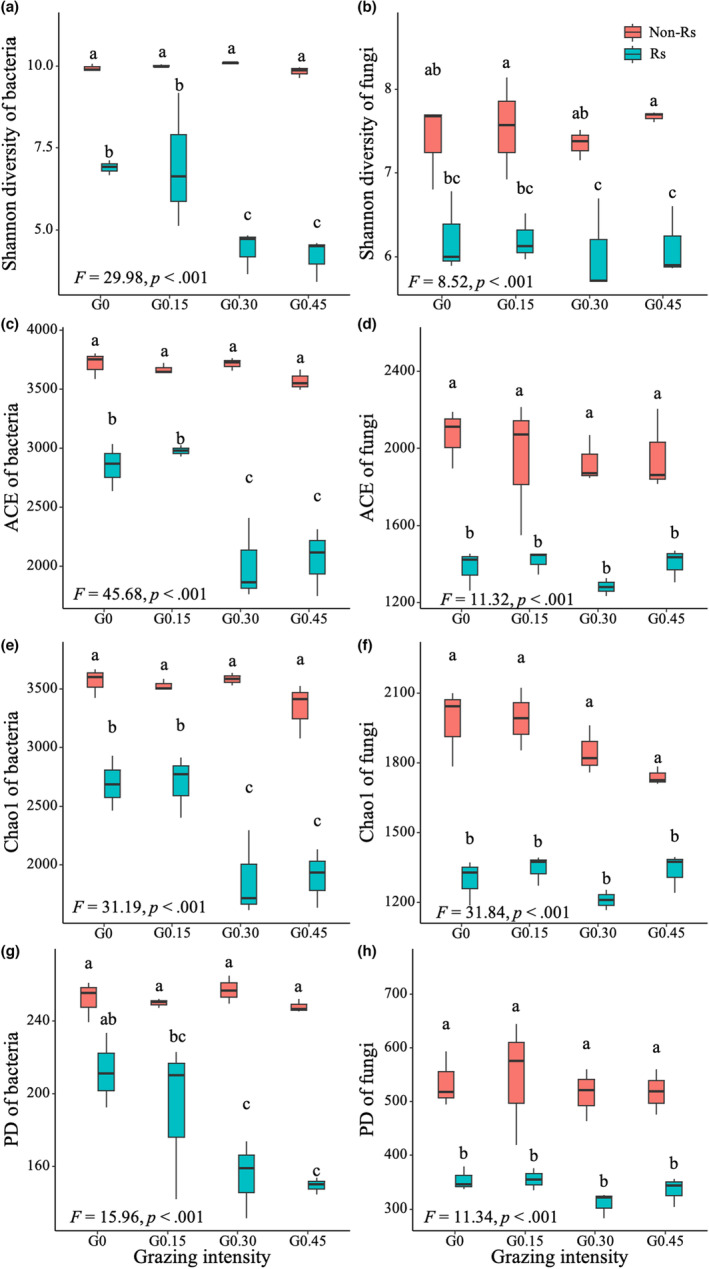
Effects of grazing intensity on soil bacterial and fungal diversity indices in rhizosphere and non‐rhizosphere soils. *Note*: G0, G0.15, G0.30, and G0.45 refer to control, light, moderate, and heavy grazing, respectively. Different lowercase letters indicate significant difference among treatments at *p* = .05.

An intriguing result from our experiment was that the rhizosphere bacterial diversity was more sensitive to grazing intensity than that in non‐rhizosphere (Zhang et al., 2019). As indicated in Figure 2a, the rhizosphere bacterial richness declined dramatically under heavy grazing intensity, while that in non‐rhizosphere remained unchanged (Zhang et al., 2019). Such differing responses also held for other diversity indices, including Shannon diversity, ACE, Chao1, and phylogenetic diversity (Figure A2). These consistent results suggest that grazing intensity has a strong impact on rhizosphere bacterial diversity. Two mechanisms may underlie the grazing‐induced decline in rhizosphere bacterial diversity. First, herbivory removes the aboveground organs of a plant, resulting in a reduction in the assimilated carbon allocated to belowground organs, and thus a decline in the quantity of carbon sources available to bacteria in the rhizosphere of the plant (Aldezabal et al., 2015; Byrnes et al., 2018; Mueller et al., 2017), which in turn reduces rhizosphere bacterial diversity. In this study, the rhizosphere bacterial richness was significantly affected by root carbon (Figure 4), providing support for this mechanism. Second, plant diversity is usually coupled with microbial diversity in a community (De Deyn & van der Putten, 2005). Previous studies have suggested that biodiversity can be enhanced by intermediate disturbance (Connell, 1978). However, in this desert steppe, long‐term grazing, even under low intensity, greatly reduced the species diversity (Zhang et al., 2021). Grazing‐induced reduction in plant diversity can decrease the diversity of carbon sources for bacterial taxa (Dwivedi et al., 2017), as a consequence, some bacterial taxa cannot grow well or disappear and the rhizosphere bacterial diversity declines.

In contrast to the response of rhizosphere bacterial richness, the fungal diversity was not significantly affected by grazing intensity in both zones (Figures 2b and A2), which is consistent with results from grazing experiments (Yang et al., 2021; Zhang & Fu, 2020). The weak response of fungal richness may be due to the fact that fungi were more tolerant to environmental stresses than bacteria (Rousk & Bååth, 2011). For example, fungi often exhibit more resistance to soil drought or acidity than bacteria (Rousk & Bååth, 2011). In this study, the fungal richness was not significantly affected by the biotic and abiotic factors examined (Figure 4), implying that the grazing‐induced changes in these variables did not exert a significant impact on the fungal richness. Overall, our results suggest that grazing on this grassland has little impact on the soil fungal richness.

**FIGURE A3:**
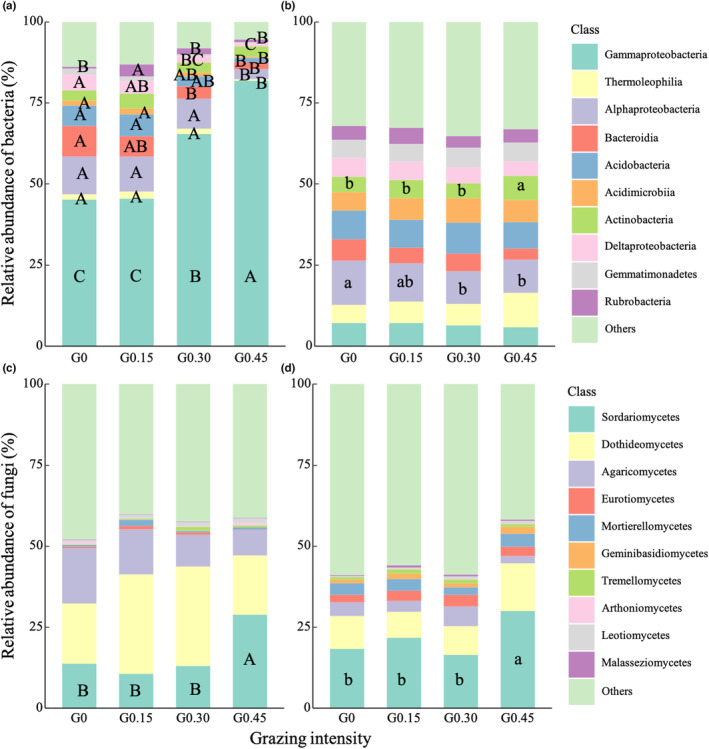
Effects of grazing intensity on the relative abundance of main soil bacterial and fungal classes in rhizosphere (a, c) and non‐rhizosphere (b, d) soils. *Note*: G0, G0.15, G0.30, and G0.45 refer to control, light, moderate, and heavy grazing, respectively. Different uppercase and lowercase letters indicate significant difference among grazing intensities in rhizosphere and non‐rhizosphere soils, respectively. The abundance of a letter indicates that the difference is not significant among grazing intensities in both soils. Significant level is *p* = .05.

**FIGURE A4:**
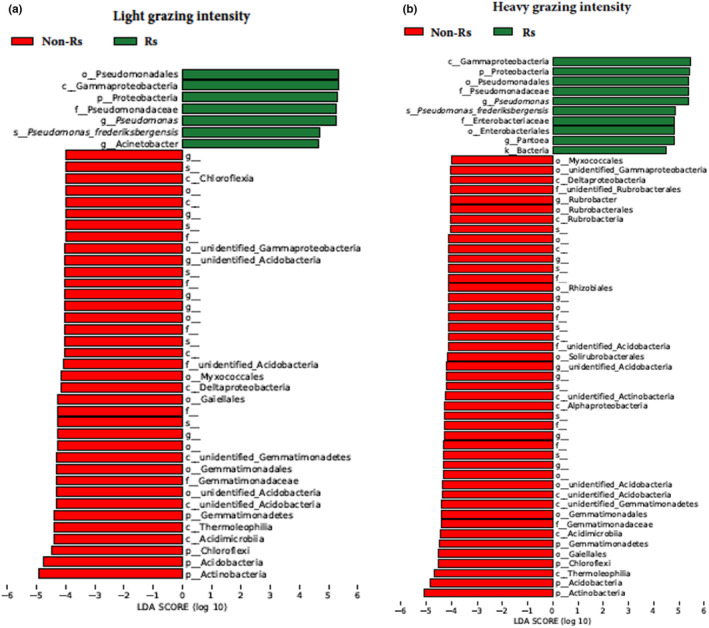
Identity biomarkers with linear discriminant analysis (LDA) scores of 4 or greater in bacterial community between rhizosphere (Rs) and non‐rhizosphere (Non‐Rs) soils under light (a) and heavy (b) grazing intensities. *Note*: LEfSe analysis (LDA ≥ 4) only detected identify biomarkers between rhizosphere and non‐rhizosphere zones under light and heavy grazing intensities, while no identify biomarkers of fungi were detected between two zones under all grazing intensities.

**FIGURE A5:**
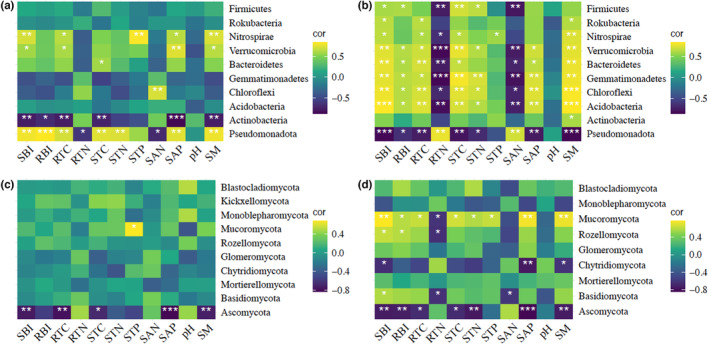
Relationship of the relative abundance of main bacterial phylum in non‐rhizosphere (a) and in rhizosphere (b) soil, main fungal phylum in non‐rhizosphere (c) and in rhizosphere (d) soil with biotic and abiotic variables. ***, *p* < .001; **, *p* < .01; *, *p* < .05. RBI, root biomass; RTC, root total carbon; RTN, root total nitrogen; SAN, soil‐available nitrogen; SAP, soil‐available phosphorus; SBI, shoot biomass; SM, soil moisture; STC, soil total carbon; STN, soil total nitrogen; STP, soil total phosphorus.

**FIGURE A6:**
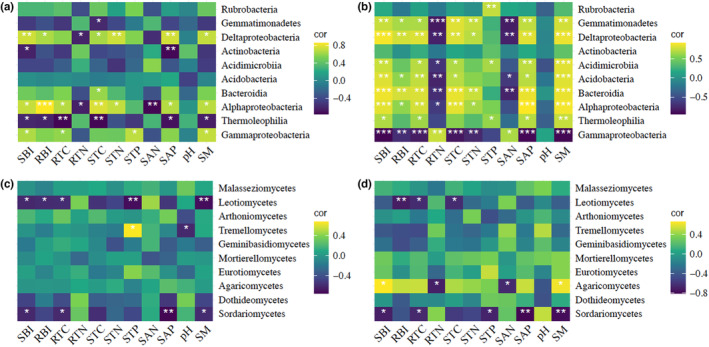
Relationship of the relative abundance of main bacterial class in non‐rhizosphere (a) and in rhizosphere (b) soil, main fungal class in non‐rhizosphere (c) and in rhizosphere (d) soil with biotic and abiotic variables. ***, *p* < .001; **, *p* < .01; *, *p* < .05. RBI, root biomass; RTC, root total carbon; RTN, root total nitrogen; SAN, soil‐available nitrogen; SAP, soil‐available phosphorus; SBI, shoot biomass; SM, soil moisture; STC, soil total carbon; STN, soil total nitrogen; STP, soil total phosphorus.

Our results suggest that grazing intensity has stronger impacts on bacterial composition in rhizosphere than that in non‐rhizosphere. For example, heavy grazing resulted in a 6.55‐fold increase in community dissimilarity in rhizosphere while a 1.89‐fold increase in non‐rhizosphere (Figure 3a). An impressive result of this experiment was that, with the increase of grazing intensity, the relative abundance of a dominant bacterial phylum, Pseudomonadota, decreased in non‐rhizosphere but increased in rhizosphere (Figure 3b,c). Such opposite responses to grazing intensity were likely due to its copiotrophic strategy in nutrient use (Leff et al., 2015). The increased grazing intensity led to a relatively higher plant root nitrogen (Tables A6 and A7), which in turn increased the relative abundance of Pseudomonadota in rhizosphere due to the positive relationship between the two (Figure A5a). By contrast, the grazing‐induced decrease in relative abundance of Pseudomonadota in non‐rhizosphere was related to the reduction in soil total nitrogen (Figure A5b; Tables A6 and A7). This was especially the case for Alphaproteobacteria, as its relative abundance was closely related to soil nitrogen (Figure A6a; Tables A6 and A7), and a significant decrease in Alphaproteobacteria was observed in non‐rhizosphere zone under moderate and heavy grazing intensities (Figure A3b; Table A5).

Our results also demonstrated strong impacts of grazing intensity on fungal composition in this grassland. As indicated by the increased dissimilarity of the fungal community with grazing intensities (Figure 3d), the fungal composition was dramatically altered by heavy grazing in both zones. A significant increase in relative abundance was observed for a dominant taxon, Ascomycota, under heavy grazing intensity (Figure 3e,f). This phenomenon could be explained by its oligotrophic strategy, which facilitates its growth in a relatively low‐carbon environment (Sterkenburg et al., 2015). As indicated in this experiment, heavy grazing resulted in a great reduction in carbon concentrations in plant roots and soils (Tables A6 and A7), and the relative abundance of Ascomycota was negatively related to these carbon concentrations (Figure A5c,d).

### 4.2 The roles of biotic and abiotic factors in affecting microbial responses to grazing intensity

In this experiment, the grazing‐induced decrease in rhizosphere bacterial richness was related to changes in 9 of 11 variables (Figure 4), consistent with a previous study in alpine meadow finding that soil microbial richness was affected by multiple biotic and abiotic factors (Zhang & Fu, 2020). Moreover, we found that all the examined biotic variables were closely related to rhizosphere bacterial richness (Figure 4), highlighting the impacts of plant selection effect on rhizosphere bacterial richness (Hartmann et al., 2009; Nan et al., 2020). However, the examined variables had little effect on fungal richness in both zones (Figure 4), consistent with previous results (Yang, Niu, et al., 2018; Yang, Zhu, et al., 2018). This may be due to the fact that fungi are more resistant to environmental variability relative to bacteria (Rousk & Bååth, 2011).

Previous studies have highlighted the role of soil pH in affecting microbial richness (Qu et al., 2016; Yang, Niu, et al., 2018; Yang, Zhu, et al., 2018; Zhang & Fu, 2020). However, in this study, the changes in bacterial and fungal richness were not affected by soil pH in both zones. The weak effect of soil pH in our study could be explained by the results that soil pH in this experiment was not significantly altered by grazing intensity (Tables A6 and A7).

Our results indicated that the bacterial and fungal compositions in both rhizosphere and non‐rhizosphere zones were co‐affected by several biotic and abiotic variables (Figure 5a). The rhizosphere bacterial composition was primarily affected by the root biomass and the root carbon concentration, highlighting the importance of plant‐related resources in affecting rhizosphere bacterial communities (Berg & Smalla, 2009; Costa et al., 2006). In contrast, the bacterial composition in non‐rhizosphere soil was primarily affected by soil‐available phosphorus, highlighting the role of abiotic factors in affecting non‐rhizosphere bacterial composition (Schöps et al., 2018; Tian et al., 2017). Moreover, soil‐available phosphorus was also the main factor that affected the fungal composition in both zones (Figure 5d,e). The important role of soil available‐phosphorus in influencing the microbial composition may be due to the fact that grasslands in northern China have relatively lower soil phosphorus than other grasslands (Han et al., 2005). For example, the soil total phosphorus concentration (515 mg kg^−1^) in this experiment site (Table A7) was much lower than the mean value (699 mg kg^−1^) in grasslands of North America (US Geological Survey, 2001). In addition, soils in this region are rich in Ca^2+^ and Mg^2+^, and phosphorus is usually bounded to these metal ions, further constraining the available phosphorus for soil fungi (Wang, Yang, et al., 2021). Importantly, the lack of soil‐available phosphorus was exacerbated by heavy grazing in this experiment (Tables A6 and A7). This is likely due to grazing‐induced losses of fertile soils. The Mongolia steppes are located in the west wind belt and are characterized by strong winds throughout the year. Years of heavy grazing caused a decrease in vegetation cover and an increase in bare land patches. As a result, strong winds caused severe wind erosion, such as sand‐dust storms, by which the nutrient‐rich soils in top layers were removed from grasslands (Giese et al., 2013). Given that some arbuscular mycorrhizal fungi can help plants efficiently exploit and absorb soil phosphorus (Faghihina et al., 2020; van der Heijden et al., 1998; van der Heyde et al., 2017), the strong relationship between soil‐available phosphorus and the fungal community composition also suggests that some fungal taxa may play an important role in mediating the availability of soil phosphorus in this grassland. Overall, our study provides experimental evidence that grazing intensity caused changes in abiotic and biotic factors, and these changes in turn altered the soil microbial composition.

## 5 CONCLUSION

Our results demonstrated that heavy grazing markedly altered the compositions of microbial communities in both rhizosphere and non‐rhizosphere soils, as well as the rhizosphere bacterial diversity. While root nitrogen and soil nitrogen played an important role in shaping the rhizosphere bacterial composition, soil‐available phosphorus greatly affected the non‐rhizosphere bacterial composition and the fungal composition in both zones. Our findings suggest that restoring and sustaining the diversity and composition of microbial communities is critical for grassland restoration in Mongolian Plateau.

## AUTHOR CONTRIBUTIONS

**Yang Yang:** Data curation (equal); formal analysis (equal); investigation (equal); writing – original draft (equal); writing – review and editing (equal). **Hao Zhang:** Investigation (equal); writing – original draft (equal); writing – review and editing (equal). **Wei Liu:** Formal analysis (equal). **Jiamei Sun:** Formal analysis (equal). **Mengli Zhao:** Conceptualization (equal). **Guodong Han:** Conceptualization (equal). **Qingmin Pan:** Project administration (equal); supervision (equal); writing – original draft (equal); writing – review and editing (equal).

## CONFLICT OF INTEREST STATEMENT

The authors declare that they have no competing interests.

### OPEN RESEARCH BADGES

This article has earned an Open Data badge for making publicly available the digitally‐shareable data necessary to reproduce the reported results. The data is available at https://github.com/IBCASyangyang/Public‐data‐for‐ECE.

## Data Availability

The data that support the findings of this study are available from the Dryad Digital Repository https://doi.org/10.5061/dryad.g4f4qrfvq.

## Appendix

**TABLE A1:** The high‐quality reads and OTU number of the rhizosphere and non‐rhizosphere soil samples under different grazing intensities.

Grazing intensity treatment	Sample	Bacteria				Fungi			
		Rhizosphere soil		Non‐rhizosphere soil		Rhizosphere soil		Non‐rhizosphere soil	
		Reads	OTU	Reads	OTU	Reads	OTUs	Reads	OTUs
G0	1	77,809	2421	80,179	3209	80,089	934	80,547	1520
G0	2	76,933	2327	80,137	3045	80,111	1054	73,825	1668
G0	3	70,741	2334	80,129	3143	80,131	978	75,929	1363
G0.15	1	77,771	2164	80,257	3129	80,161	1035	76,083	1728
G0.15	2	78,841	2282	80,155	3166	80,114	1016	78,991	1529
G0.15	3	82,925	2202	80,145	3065	80,230	948	73,687	1357
G0.30	1	82,478	1417	80,211	3247	80,128	971	80,079	1280
G0.30	2	80,041	1663	80,252	3116	80,138	923	76,193	1584
G0.30	3	80,097	1683	80,199	3205	80,128	865	82,381	1291
G0.45	1	80,096	1418	76,193	3010	80,158	1014	83,547	1410
G0.45	2	80,112	1456	80,178	3044	80,095	1029	72,338	1379
G0.45	3	80,143	1793	80,229	2899	80,169	928	74,690	1315
Total	24	947,987	23,160	958,264	37,278	961,649	11,695	928,290	17,424

**TABLE A2:** The relative abundances of main bacterial and fungal phyla under different grazing intensities in rhizosphere and non‐rhizosphere zones.

	Phylum	Rhizosphere soil				Mean	Non‐rhizosphere soil				Mean
		Grazing intensity treatment					Grazing intensity treatment				
		G0	G0.15	G0.30	G0.45		G0	G0.15	G0.30	G0.45	
Bacteria	Proteobacteria	61.81	67.14	83.94	84.66	74.39	27.51	24.04	21.55	21.45	23.64
	Acidobacteria	11.56	8.65	3.28	2.27	6.44	21.82	20.62	22.66	19.68	21.19
	Actinobacteria	6.06	8.25	4.70	4.46	5.87	22.95	26.73	24.58	32.40	26.67
	Bacteroidetes	8.02	4.42	3.64	2.27	4.59	5.37	5.06	5.81	3.67	4.98
	Chloroflexi	3.37	3.39	0.60	1.20	2.14	8.41	8.98	10.12	9.07	9.15
	Gemmatimonadetes	1.98	2.24	0.63	0.45	1.33	6.83	6.42	7.55	7.04	6.96
Fungi	Ascomycota	40.72	46.62	48.57	59.08	48.75	31.07	33.75	38.07	51.60	38.62
	Basidiomycota	21.88	11.08	14.35	7.60	13.73	5.57	7.43	13.41	5.73	8.04

*Note*: Main bacterial and fungal phyla indicate the phyla with relative abundance higher than 1%.

**TABLE A3:** The relative abundances of main bacterial and fungal class under different grazing intensities in rhizosphere and non‐rhizosphere zones.

	Taxonomy	Rhizosphere soil				Mean	Non‐rhizosphere soil				Mean
		Grazing intensity treatment					Grazing intensity treatment				
		G0	G0.15	G0.30	G0.45		G0	G0.15	G0.30	G0.45	
Bacteria	Gammaproteobacteria	45.22	45.54	65.42	81.82	59.50	7.12	7.06	6.42	5.78	6.59
	Thermoleophilia	1.58	2.02	1.61	0.41	1.41	5.58	6.67	6.55	10.67	7.37
	Alphaproteobacteria	11.70	10.85	9.18	3.19	8.73	13.73	11.74	10.13	10.16	11.44
	Bacteroidia	9.41	6.30	3.98	2.13	5.45	6.52	4.83	5.43	3.42	5.05
	Acidobacteria	6.23	6.69	2.92	1.34	4.30	8.84	8.68	9.57	8.17	8.82
	Acidimicrobiia	1.57	1.88	1.28	0.44	1.29	5.71	6.70	7.57	6.82	6.69
	Actinobacteria	3.16	4.58	3.07	3.06	3.47	4.70	5.52	4.51	7.41	5.53
	Deltaproteobacteria	4.86	3.78	1.98	0.94	2.89	5.89	5.74	4.99	4.45	5.27
	Gemmatimonadetes	1.86	1.46	0.60	0.39	1.08	5.54	5.32	6.03	5.90	5.70
	Rubrobacteria	0.51	3.79	1.77	0.88	1.74	4.30	5.03	3.50	4.09	4.23
Fungi	Sordariomycetes	13.75	10.67	12.99	23.83	16.56	18.36	21.76	16.45	30.06	21.66
	Dothideomycetes	18.57	30.60	30.75	18.29	24.55	10.13	7.98	8.81	14.70	10.41
	Agaricomycetes	17.09	13.93	9.85	7.78	12.16	4.22	3.36	6.19	2.26	4.01
	Eurotiomycetes	0.39	1.06	0.69	0.2	0.58	2.23	3.17	3.54	2.76	2.93
	Mortierellomycetes	0.57	1.88	0.42	0.66	0.88	3.58	3.64	2.33	3.99	3.38
	Geminibasidiomycetes	0.002	0.09	0.12	0.05	0.06	1.15	1.57	1.24	2.13	1.52

*Note*: Main bacterial and fungal class indicate the class with relative abundance higher than 1%.

**TABLE A4:** Results (*F* and *p* values) of one‐way ANOVA of the effects of grazing intensity on the relative abundance of main bacterial and fungal phyla in rhizosphere and non‐rhizosphere zones.

	Phylum	Rhizosphere		Non‐rhizosphere	
		*F* value	*p* Value	*F* value	*p* Value
Bacteria	Proteobacteria	130.20	**<.001**	12.43	**.002**
	Acidobacteria	97.71	**<.001**	9.00	**<.001**
	Actinobacteria	4.90	**.03**	36.88	**<.001**
	Chloroflexi	16.10	**<.001**	16.10	.09
	Gemmatimonadetes	41.95	**<.001**	2.29	.16
	Bacteroidetes	60.04	**<.001**	5.62	**.023**
Fungi	Ascomycota	180.4	**<.001**	68.73	**<.001**
	Basidiomycota	53.32	**<.001**	13.59	**.002**
	Mortierellomycota	1.35	.33	1.51	.29
	Chytridiomycota	5.67	**.022**	0.26	.86

*Note*: Significant effects are highlighted in bold (*p* < .05). The soil zones refer to plant rhizosphere and non‐rhizosphere.

**TABLE A5:** Results (*F* and *p* values) of one‐way ANOVA of the effects of grazing intensity on the relative abundance of main bacterial and fungal classes in rhizosphere and non‐rhizosphere zones.

	Phylum	Rhizosphere		Non‐rhizosphere	
		*F* value	*p* Value	*F* value	*p* Value
Bacteria	Gammaproteobacteria	39.95	**<.001**	1.15	.388
	Thermoleophilia	11.99	**.002**	3.83	.057
	Alphaproteobacteria	13.44	**.002**	5.95	**.019**
	Bacteroidia	12.94	**.002**	1.65	.253
	Acidobacteria	9.43	**.005**	1.63	.258
	Acidimicrobiia	9.90	**.004**	1.30	.341
	Actinobacteria	1.86	.215	12.28	**.002**
	Deltaproteobacteria	16.11	**<.001**	3.35	.076
	Gemmatimonadetes	8.93	**.006**	1.25	.354
	Rubrobacteria	30.15	**<.001**	2.21	.165
Fungi	Sordariomycetes	16.91	**<.001**	20.21	**<.001**
	Dothideomycetes	4.02	.051	3.10	.089
	Agaricomycetes	2.65	.12	1.30	.34
	Eurotiomycetes	2.54	.13	0.24	.87
	Mortierellomycetes	1.23	.359	1.54	.285
	Geminibasidiomycetes	0.86	.502	0.28	.842
	Tremellomycetes	0.98	.448	0.68	.587
	Arthoniomycetes	0.66	.599	0.80	.528
	Leotiomycetes	0.82	.519	1.69	.245
	Malasseziomycetes	1.22	.363	0.65	.605

*Note*: Significant effects are highlighted in bold (*p* < .05). The soil zones refer to plant rhizosphere and non‐rhizosphere.

**TABLE A6:** Results (*F* and *p* values) of one‐way ANOVA of the effects of grazing intensity on biotic and abiotic variables.

Variables	*F* value	*p* Value
Shoot biomass (g individual^−1^)	38.360	.001
Root biomass (g individual^−1^)	40.740	<.001
Root total carbon (g kg^−1^)	12.871	.003
Root total nitrogen (g kg^−1^)	11.477	.004
Soil total carbon (g kg^−1^)	7.002	.016
Soil total nitrogen (g kg^−1^)	9.509	.007
Soil total phosphorus (g kg^−1^)	7.661	.013
Soil‐available nitrogen (mg kg^−1^)	130.889	<.001
Soil‐available phosphorus (mg kg^−1^)	46.030	<.001
pH value	1.023	.438
Soil moisture (%)	38.470	.001

**TABLE A7:** Biotic and abiotic variables under different grazing intensities.

Variables	Grazing intensity treatment			
	G0	G0.15	G0.30	G0.45
Shoot biomass (g individual^−1^)	5.25 ± 0.32 c	4.37 ± 0.48 c	2.42 ± 0.29 b	0.80 ± 0.08 a
Root biomass (g individual^−1^)	6.08 ± 0.28 c	4.54 ± 0.18 c	3.75 ± 0.18 b	3.46 ± 0.54 a
Root total carbon (g kg^−1^)	431.46 ± 1.38 c	425.66 ± 2.22 bc	415.93 ± 3.41 ab	404.29 ± 7.42 a
Root total nitrogen (g kg^−1^)	21.24 ± 0.99 a	21.88 ± 0.56 a	24.39 ± 0.25 b	24.61 ± 0.52 b
Soil total carbon (g kg^−1^)	15.80 ± 0.43 b	15.08 ± 0.50 ab	13.65 ± 0.20 ab	12.64 ± 0.77 a
Soil total nitrogen (g kg^−1^)	1.88 ± 0.04 b	1.80 ± 0.04 ab	1.67 ± 0.02 a	1.66 ± 0.03 a
Soil total phosphorus (g kg^−1^)	0.51 ± 0.01 ab	0.55 ± 0.00 b	0.51 ± 0.02 ab	0.49 ± 0.01 a
Soil‐available nitrogen (mg kg^−1^)	26.99 ± 0.31 a	35.97 ± 1.01 b	45.87 ± 0.80 c	42.38 ± 1.14 d
Soil‐available phosphorus (mg kg^−1^)	4.08 ± 0.08 c	3.70 ± 0.18 bc	3.44 ± 0.02 b	2.59 ± 0.03 a
pH value	7.82 ± 0.02	7.72 ± 0.07	7.73 ± 0.10	7.86 ± 0.03
Soil moisture (%)	3.05 ± 0.06 c	3.02 ± 0.08 c	2.40 ± 0.12 b	1.99 ± 0.06 a

*Note*: Different letters indicate the significant differences among treatments. Values are the means ± SE. Values with different letters indicate significant difference among treatments at *p* = .05.
